# Selection of suitable candidate genes for mRNA expression normalization in bulbil development of *Pinellia ternata*

**DOI:** 10.1038/s41598-022-12782-5

**Published:** 2022-05-25

**Authors:** Haoyu Fan, Qiuling He, Yiheng Dong, Wenxin Xu, Yanlin Lou, Xuejun Hua, Tao Xu

**Affiliations:** Key Laboratory of Plant Secondary Metabolism and Regulation of Zhejiang Province, Zhejiang Sci-Tech University, Hangzhou, China

**Keywords:** Plant molecular biology, Transcriptomics

## Abstract

*Pinellia ternata* (Thunb.) Breit. (Abbreviated as *P*. *ternata*). It is a commonly prescribed Chinese traditional medicinal herb for the treatment of phlegm, cough, and morning sick. Bulbil reproduction is one of the main reproductive methods of *P*. *ternata.* The accurate quantification of gene expression patterns associated with bulbil development might be helpful to explore the molecular mechanism involved in *P*. *ternata* reproduction. Quantitative real-time PCR was the most preferred method for expression profile and function analysis of mRNA. However, the reference genes in different tissues of *P. ternata* in different periods of bulbil development have not been studied in detail. In present study, the expression stability of eight candidate reference genes were determined with programs: geNorm, NormFinder, BestKeeper, and refFinder. Glyceraldehyde-3-phosphate dehydrogenase (*GAPDH*) was identified as the top- rated reference gene in all samples of *P. ternata*, while different combinations of reference gene proved to be the most stable depending on development stage and tissue type. Furthermore, the reliability of *GAPDH* expression was verified by six *P*. *ternata* related genes in hormone and nutrient biosynthesis pathways, and the expression profiles of these genes were agreed with the results of RNA-seq digital gene expression analysis. These results can contribute to studies of gene expression patterns and functional analysis of *P*. *ternata* involved in bulbil development.

## Introduction

*Pinellia ternata* (Thunb.), belongs to the Araceae family, is mainly distributed in China, Japan and Korea^1^. The dried tuber of *P*. *ternata* that called *Pinellia rhizome* (PR) can be used as medicinal materials after processing to reduce its toxicity. Pharmacological investigations have demonstrated that the chemical constituents of *P. ternata* extract contains various biological activities, such as sedative, cytotoxic, anti-tumor, hypnotic, antiemetic and anticonvulsant activities^2,3^. In recent years, the increasing demand of *P*. *ternata* has made the wild resources decreased sharply, and the problem of continuous cropping obstacle has not been effectively solved, leading to the shortage of market resources^3^. The reproductive methods of *P. ternata* include seed, tuber and bulbil. The offspring produced by asexual reproduction of bulbils can stably inherit the excellent traits of the parent plant, have the characteristics of high germination rate and non-dormancy, which provide a safe strategy for asexual reproduction^4,5^.

Gene expression analysis is an important technique for elucidation of biological genetics, signaling, metabolic pathways, and complex regulatory networks for biotic and abiotic stress responses^6^.The development of sequencing technology provides a large amount of sequence information of plant species, which makes the study of plant gene expression more frequent and convenient^7^. qRT-PCR is an important tool for efficient, sensitive, and reliable analysis of gene expression level in biological samples^8^. However, the researchers found that the results of qRT-PCR are often affected by various factors, such as RNA quality, reverse transcription efficiency, amplification efficiency^9^. In the process of data processing, the experimental data needed to be calibrated^9,10^. The most commonly used method for calibrating target expression is to use internal control genes as a reference, namely reference genes (RGs). The important characteristic of the ideal reference gene is that the gene expression level is stable in different experimental conditions, different tissues and different developmental stages^11^. The commonly used RGs include *18SrRNA*, *β-actin*, *GAPDH*, *EF1-α*, *α-tubulin* and *β-tubulin*^11–16^. However, researchers have found that there are no ideal RGs that can be stably expressed under any experimental conditions. Thus, it is necessary to screen the best RGs according to different plant species, tissues or various experimental conditions^17^. RGs have been reported in many plant species, such as the model plant *Arabidopsis thaliana*^18^, *Oryza sativa*^19^, *Glycine max*^20^. Furthermore, the identification of RGs have been reported in many medicinal plants, such as *Scutellaria baicalensis*^21^, *Dendrobium officinale*^22^ and *Glycyrrhiza*^8^. Screening suitable RGs has greatly facilitated functional studies of plant growth and development, but the research on the screening of RGs in *P. ternata* has not been reported.

**Figure 1:**
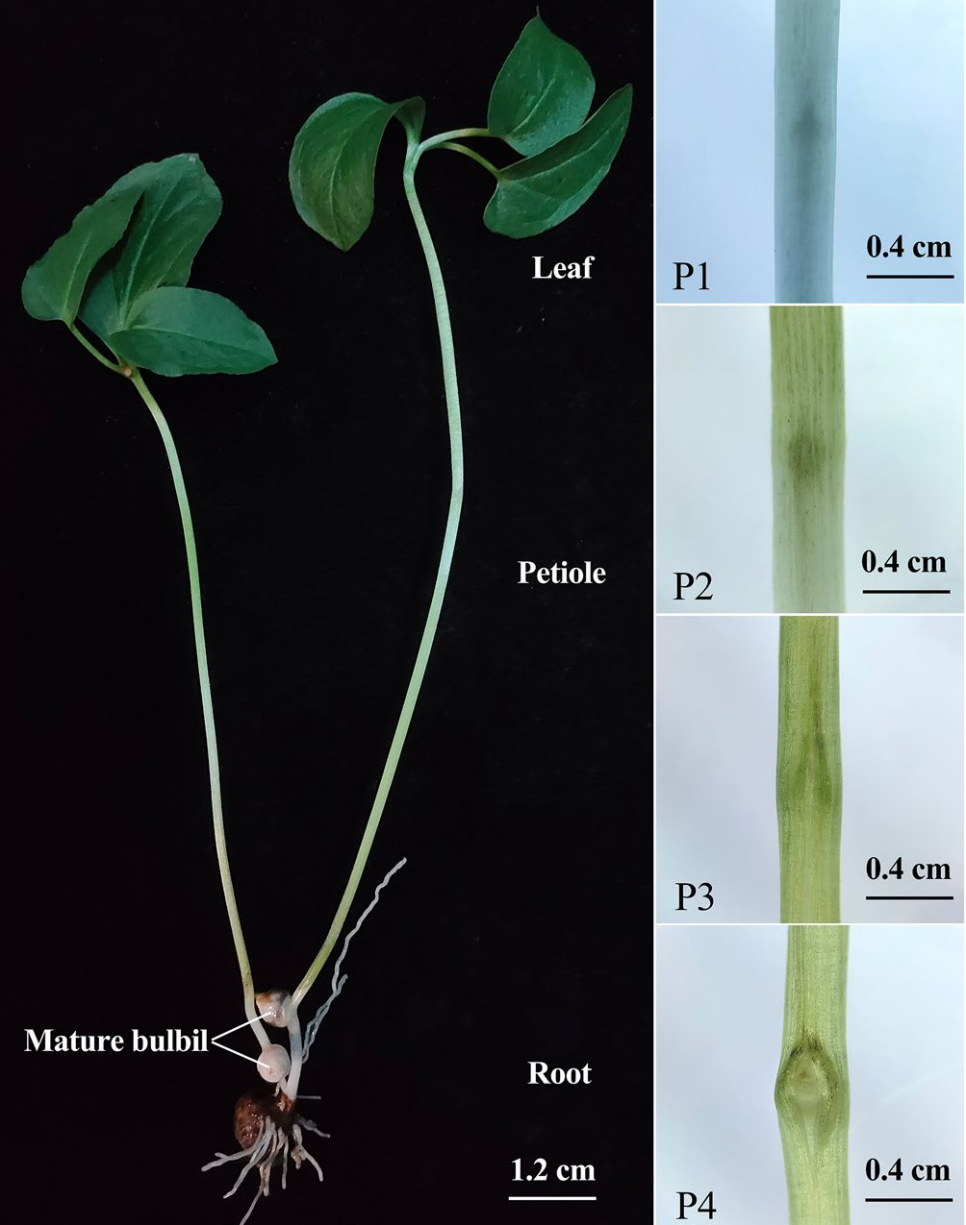
Four stages of early bulbil development and mature bulbil. (**a**). Intact plants and mature bulbil. (**b**). Four periods of sampling: P1 = Period 1; P2 = Period 2; P3 = Period 3; P4 = Period 4.

Based on published reference gene screening and their application in other plant species, 8 candidate reference genes (CRGs) were selected from *P. ternata* transcriptome database generated by previous studies of our group for this study. These CRGs were investigated to determine the most suitable candidate gene(s) as the reference(s) for gene expression analysis using qRT-PCR technique in different developmental stages and including different tissues of *P. ternata* (Fig. 1). These genes include elongation factor β subunit *(EF1-beta*), nicotinic phosphate ribose transferase (*NAPRT*), large subunit ribosomal protein L25 (*L25*), phosphoenolpyruvate carboxykinase (*PEPCK*), glyceraldehyde-3-phosphate dehydrogenase (*GAPDH*), tubulin β (*TUB*), elongation factor α subunit (*EF1-alpha*) and ADP ribosylation factor 8b (*ARL8B*). The average cycle threshold (Ct) values of CRGs were used to determine the gene expression stability using four statistical software tools: geNorm^23^, NormFinder^24^, Bestkeeper^25^, and refFinder^26^. This study screened and validated the best RGs will help researchers to improve the accuracy and reliability of gene expression analysis for qRT-PCR analysis of bulbil development in *P. ternata*, and laid a foundation for revealing the molecular mechanism related to the development of *P. ternata* in the future.

## Results

### Candidate reference genes screening and primer design

Refer to the RGs commonly used in the research of plant gene expression, four commonly used CRGs and four new genes were selected. Homologous sequences of these CRGs in *P. ternata* identified through BLASTX against GenBank which obtained from our unpublished *P. ternata* transcriptomic data. According to the identity of the annotation of each gene with its ortholog, we selected eight candidate genes including those encoding elongation factor β subunit (*EF1-beta*), nicotinic phosphate ribose transferase (*NAPRT*), large subunit ribosomal protein L25 (*L25*), phosphoenolpyruvate carboxykinase (*PEPCK*), glyceraldehyde-3-phosphate dehydrogenase (*GAPDH*), tubulin β (*TUB*), elongation factor α subunit (*EF1-alpha*) and ADP ribosylation factor 8b (*ARL8B*). Among the eight CRGs, commonly used RGs and new CRGs account for half of each (Table 1).

**Table 1:** Delineations of eight candidate reference genes in *P. ternata* and parameters gained by qRT-PCR analysis.

Gene symbol	Homolog locus	Annotation	Primer sequences (forward/reverse, 5′–3′)	Efficiencies (E %)	Product size (bp)	R^2^
*EF1-beta*	XM_015791225	Elongation factor EF-1 beta subunit	TGGGATGACGAGACGGACAT GTTGCAGGGCTCAACTGTCA	106.0	198	0.949
*NAPRT*	XM_015780447	Nicotinate phosphoribosyltransferase	ACCACTTCGTGGAACCCATT TCTCTCCAAAAATGCCACGGA	90.6	182	0.999
*L25*	XM_015772552	Large subunit ribosomal protein L25	GACAGCAAGCAGATCGTCAC CCACGCAAGGACCAAGTTCA	115.0	180	0.961
*PEPCK*	XM_015775203	Phosphoenolpyruvate carboxykinase (ATP)	TCTGGCATGCGATGCATTTG TTCGTGGGGTGCAGCATTAT	95.0	183	0.990
*GAPDH*	XM_015772221	Lyceraldehyde-3-phosphate dehydrogenase	ACTGTTGATGGACCTTCTG TTGGAACTCGGAATGACAT	100.6	151	0.998
*TUB*	XM_015765391	Tubulin beta	AACAACGTCAAGTCCAGCGT CTCGGTGAACTCCATCTCGT	93.9	192	0.999
*ARL8B*	XM_015775919	ADP-ribosylation factor-like 8b	GTACCCCCGCATCCATTTCA CCACGGTACATCAGACAGCA	114.0	180	0.943
*EF1-alpha*	XM_015774317	Elongation factor EF-1 alpha subunit	GGGCGACAACATGATCGAGA GGTTTCAACACGACCAACGG	109	181	0.971

### Validation of primers

**Figure 2:**
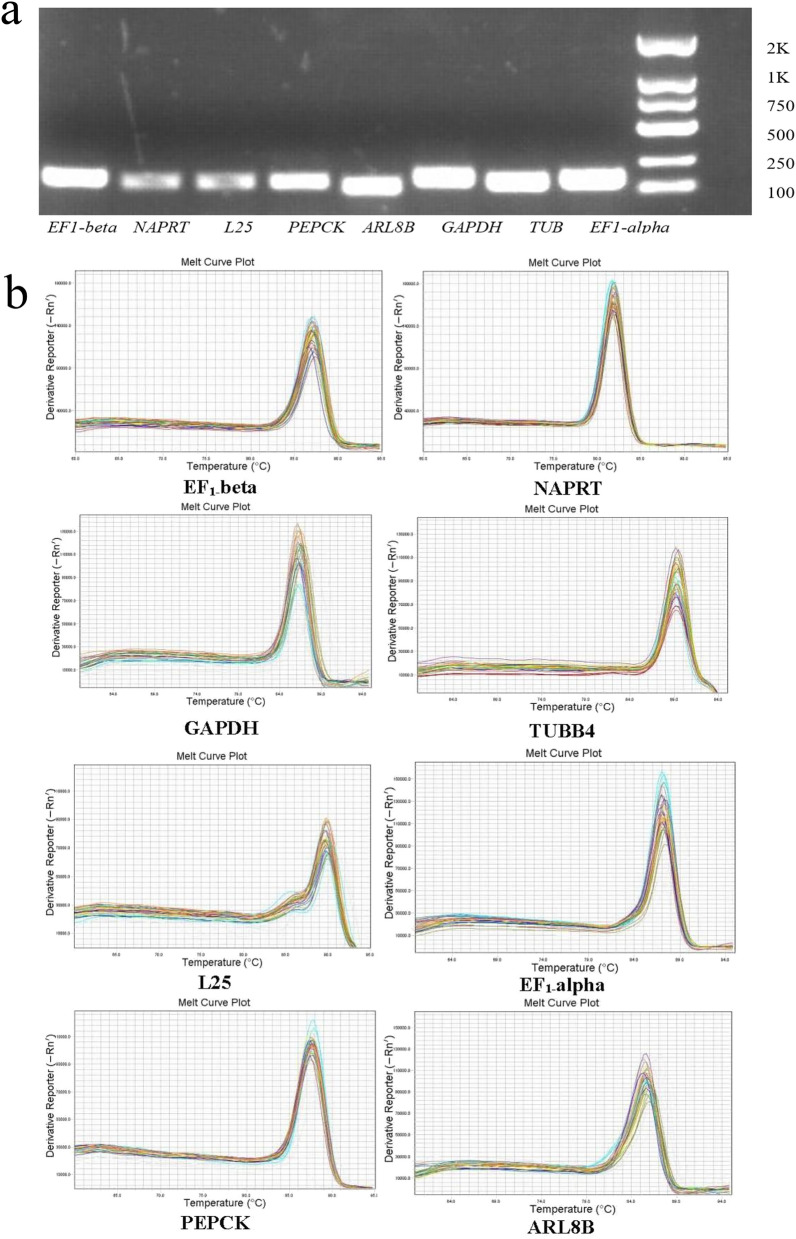
Specificity of qRT-PCR amplification. (**a**) Agarose gel (1%) showing amplification of a single product of expected size for each candidate reference gene. (**b**) Melt curves of eight candidate reference genes.

The PCR amplification specificities of primers were confirmed by agarose gel electrophoresis with single bands of the expected size, with no primer-dimer being observed. (Fig. 2a). The melting curve of each product has a single peak, which proves the specificity of primers (Fig. 2b). Amplification efficiency (E) of PCR reactions varied between 90.6 and 115.0 and the regression coefficient (R^2^) ranged from 0.943 to 0.999 (Table 1), indicated that primers of each gene exhibited significant specificity and high amplification efficiency.

### Expression profiles of candidate reference genes

**Figure 3:**
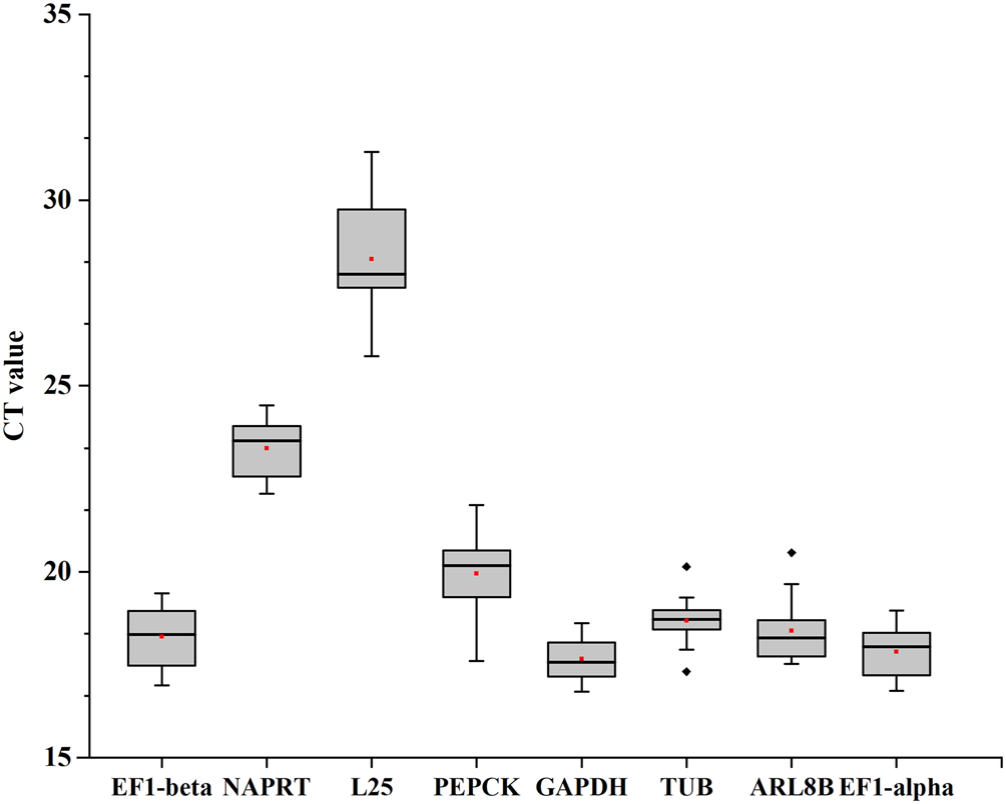
Expression levels of different candidate reference genes. Expression data displayed as qRT-PCR quantification cycle values for each reference gene in *P. ternata.* The line across the box is depicted as the median. The box indicates the 25th and 75th percentiles and whisker caps represent the maximum and minimum values. The higher the boxes and whisker are, the greater the variations.

The expression profiles of each candidate reference gene were determined by qRT-PCR during different development stages and in different tissues. The expression abundance of each gene varied across the samples of *P. ternata*. The average Ct values derived from three biological repetitions of the eight CRGs ranged from 17 to 29. The results indicated that the expression of *GAPDH* had the highest expression abundance in all the samples, with an average Ct value of 17.654, while *L25* showed the lowest expression abundance, with the Ct values being 25.8 of minimum and 31.3 of maximum. The Ct values of *GAPDH* (3.23 ± 0.57) and *TUB* (3.56 ± 0.66) with minimum SD values indicated that the stability of these genes was the best of all the candidates. CRGs with more variable expression levels included *L25* and *PCKA.* (Fig. 3, Table S1).

### Analysis of candidate reference genes expression stability

The average Ct values derived from three biological replicates of the eight candidate genes were used to evaluate stability for three evaluation methods, and refFinder was used to integrate the results of the above three methods to get the comprehensive index ranking.

**Figure 4:**
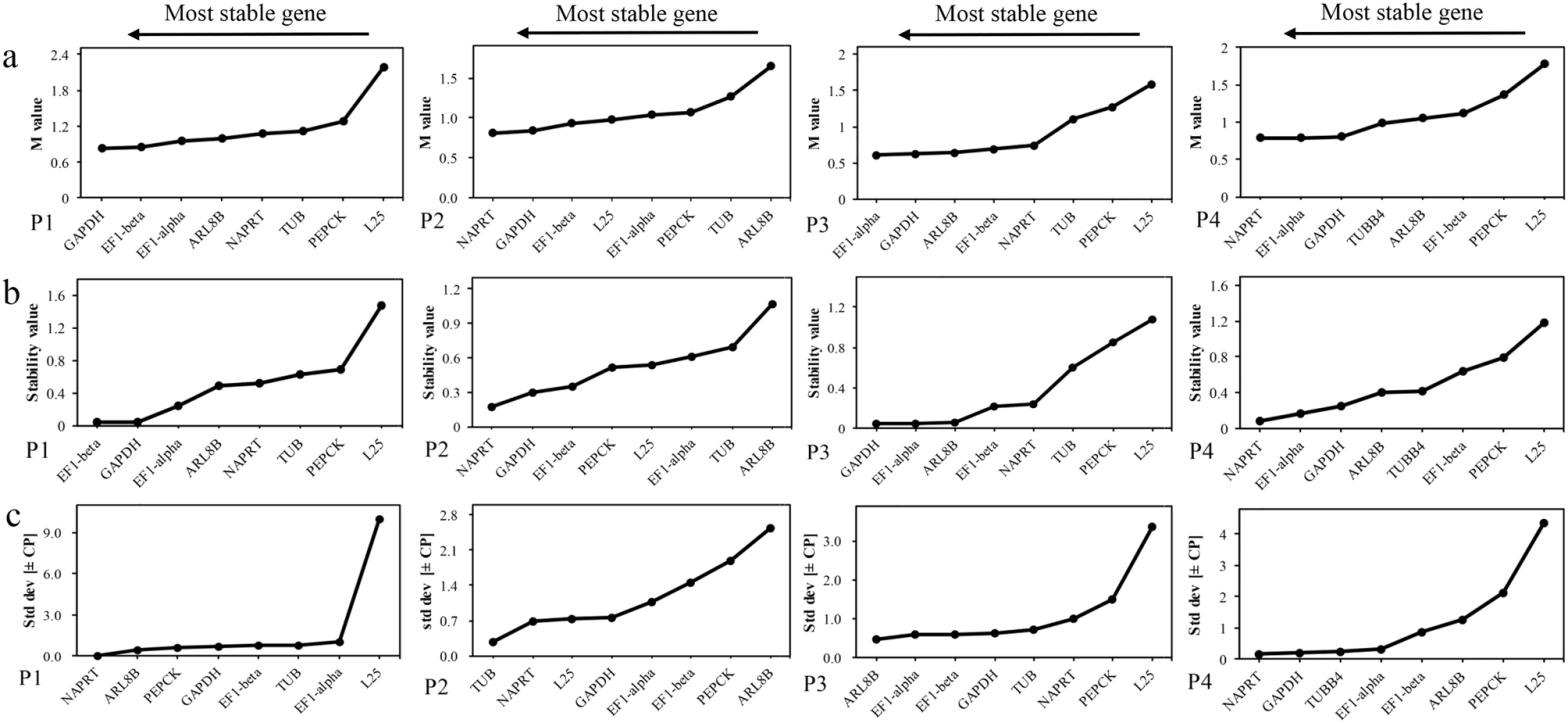
Expression stability of eight candidate reference genes in Dp of *P. ternata* as calculated by geNorm (**a**), NormFinder (**b**) and Bestkeeper (**c**)**.** P1 = Period 1; P2 = Period 2; P3 = Period 3; P4 = Period 4.

**Figure 5:**
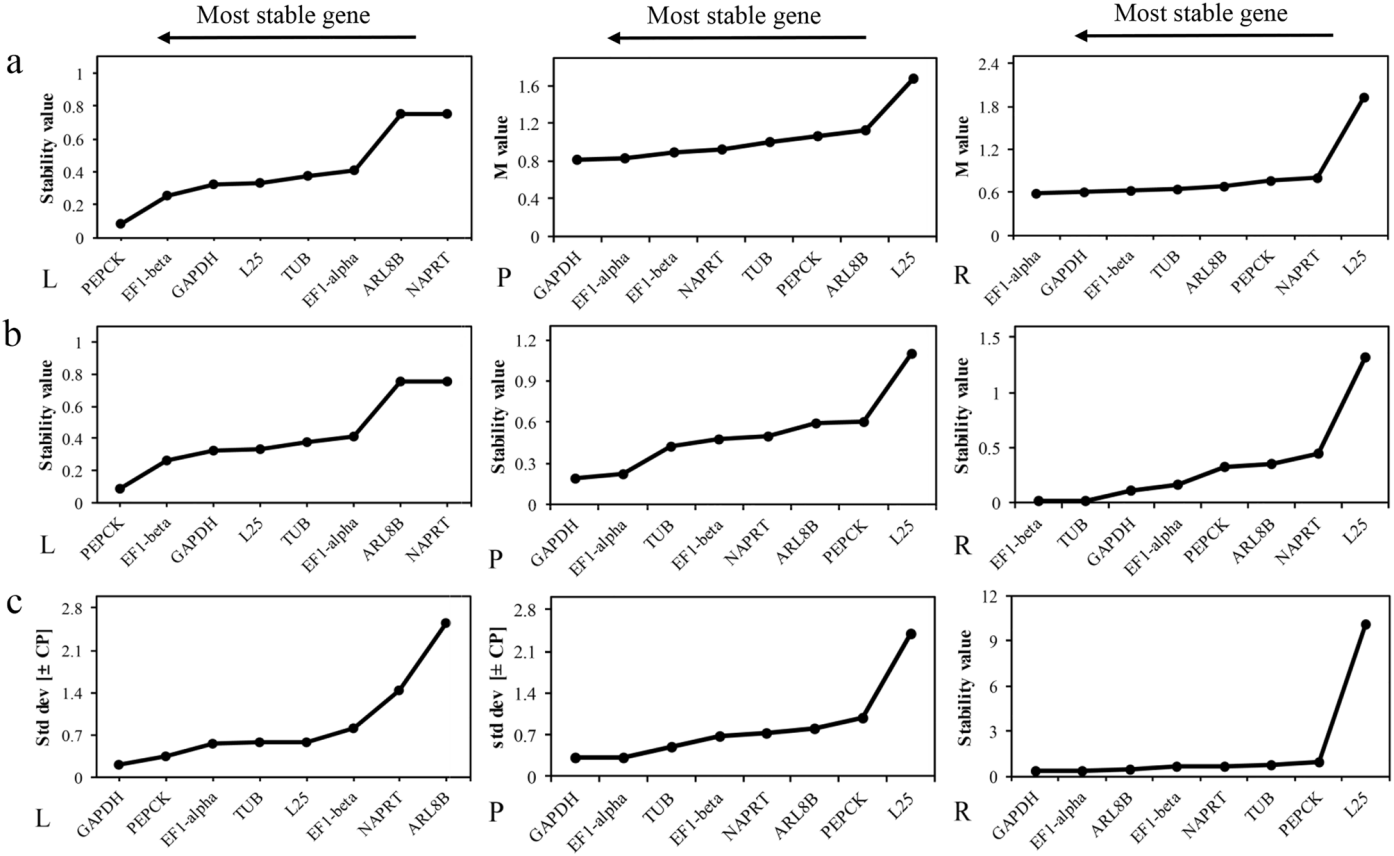
Expression stability of eight candidate reference genes in Dt of *P. ternata* as calculated by geNorm (**a**), NormFinder (**b**) and Bestkeeper (**c**). L = Leaf; P = Petiole; R = Root.

**Figure 6:**
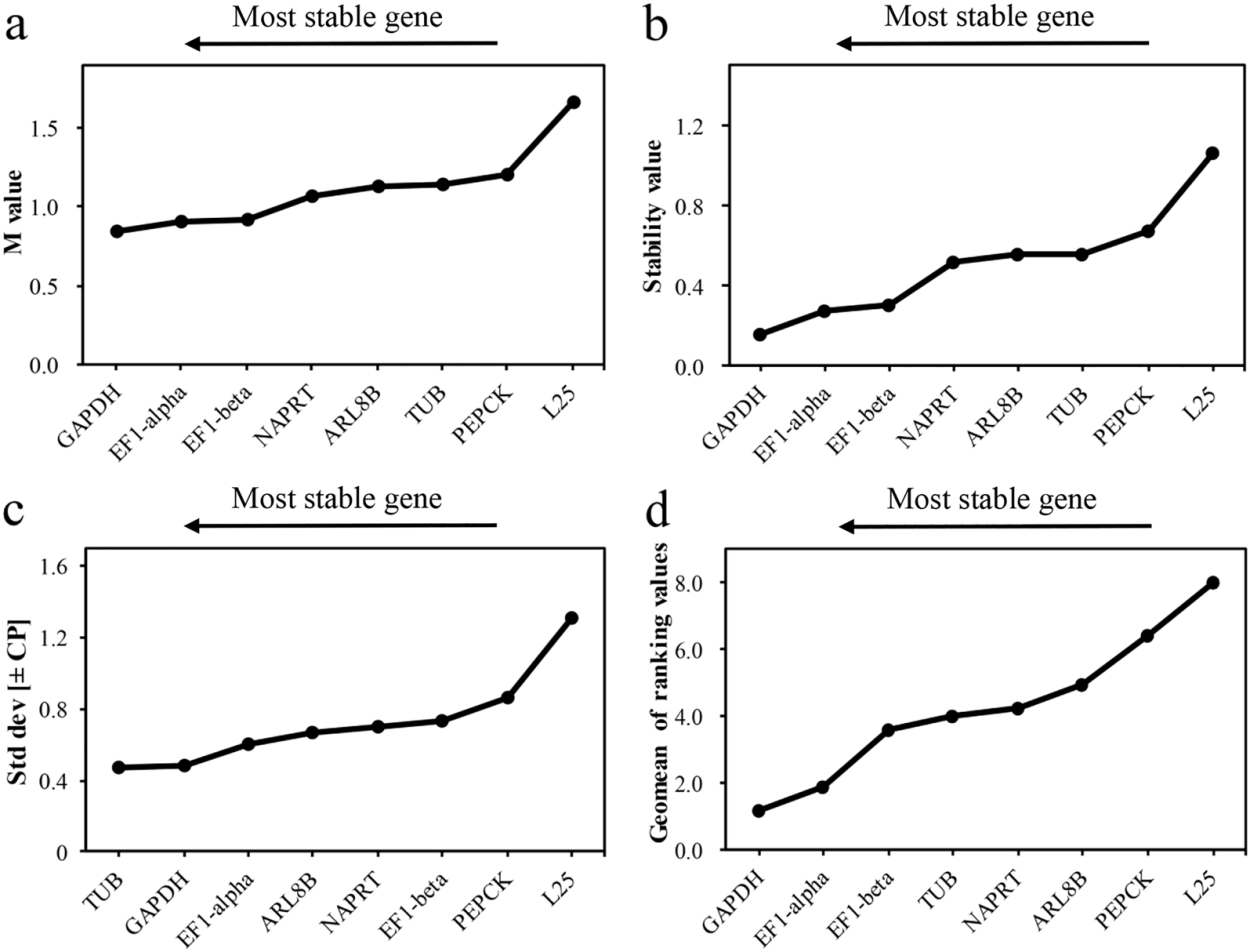
Expression stability of eight candidate reference genes in total of *P. ternata* as calculated by GeNorm (**a**), NormFinder (**b**), BestKeeper (**c**) and refFinder (**d**).

M-value was calculated by geNorm algorithm, the gene with the lowest M value was the best reference gene^27^. The results of analysis showed that, for DP, most genes showed high stability, with an M value far below the default limit of 1.5. (Fig. 4a, Table S2). However, *L25* gene had the lowest stability in period 1, 3 and 4, and *ARL8B* at 1.64 in period 2 exceeded the threshold (Fig. 4a, Table S2). The calculation showed that for different tissues, the most stable genes are *GAPDH* (0.84) and followed by *EF1-alpha* (0.90) and *EF1-beta* (0.92) (Fig. 5a, Table S2). The evaluation results of geNorm showed that *L25* exhibited low stability in all samples (Fig. 6a, Table S2).

NormFinder analyzed the expression stability of all CRGs mainly based on the results of variance analysis and ranked them in accordance with their stability^24^. In different development periods, *GAPDH* was the most stable gene followed by *NAPRT* exhibited more stable expression in Period 2 and 4 (Fig. 4b, Table S3). *EF1-beta* and *GAPDH* were the most stable genes in Dt (Fig. 5b, Table S3). In all samples, the more stable gene is *GAPDH* (0.16), followed by *EF1-alpha* (0.28) and *EF1-beta* (0.31), and the most unstable gene was *L25* (1.059) (Fig. 6b, Table S3). The order of gene expression stability from high to low was *GAPDH*, *EF-alpha*, *EF1-beta*, *NAPRT*, *ARL8B*, *TUB*, *PEPCK*, *L25* (Table 2).

**Table 2:** Expression stability of the candidate reference genes in samples of *P. ternata* as calculated by geNorm, NormFinder, BestKeeper and RefFinder.

Rank	GeNorm			NormFinder			Bestkeeper			RefFinder		
	Dp	Dt	All samples	Dp	Dt	All samples	Dp	Dt	All samples	Dp	Dt	All samples
1	*GAPDH*	*GAPDH*	*GAPDH*	*GAPDH*	*EF1-beta*	*GAPDH*	*NAPRT*	*GAPDH*	*TUB*	*GAPDH*	*GAPDH*	*GAPDH*
2	*NAPRT*	*EF1-beta*	*EF1-alpha*	*NAPRT*	*GAPDH*	*EF1-alpha*	*TUB*	*EF1-alpha*	*GAPDH*	*NAPRT*	*EF1-alpha*	*EF1-alpha*
3	*EF1-alpha*	*EF1-alpha*	*EF1-beta*	*EF1-beta*	*TUB*	*EF1-beta*	*ARL8B*	*TUB*	*EF1-alpha*	*EF1-beta*	*EF1-beta*	*EF1-beta*
4	*EF1-beta*	*PEPCK*	*NAPRT*	*EF1-alpha*	*PEPCK*	*NAPRT*	*GAPDH*	*EF1-beta*	*ARL8B*	*ARL8B*	*PEPCK*	*TUB*
5	*ARL8B*	*TUB*	*ARL8B*	*ARL8B*	*EF1-alpha*	*ARL8B*	*EF1-alpha*	*PEPCK*	*NAPRT*	*TUB*	*TUB*	*NAPRT*
6	*TUB*	*L25*	*TUB*	*TUB*	*ARL8B*	*TUB*	*EF1-beta*	*ARL8B*	*EF1-beta*	*EF1-alpha*	*ARL8B*	*ARL8B*
7	*L25*	*NAPRT*	*PEPCK*	*PEPCK*	*L25*	*PEPCK*	*PEPCK*	*NAPRT*	*PEPCK*	*L25*	*NAPRT*	*PEPCK*
8	*PEPCK*	*ARL8B*	*L25*	*L25*	*NAPRT*	*L25*	*L25*	*L25*	*L25*	*PEPCK*	*L25*	*L25*

All samples are a combination of samples from different tissues (Dt) and different periods (Dp).

The excel-based program BestKeeper was used to rank the candidate genes, the lower the SD value, the higher the stability of gene expression^25^. *NAPRT* is the most stable gene expressed in Dp (Fig. 4c, Table S4), while *GAPDH* is the most suitable genes in Dt (Fig. 5c, Table S4). For all samples, the most stable gene was *TUB* (0.47), followed by *GAPDH* (0.48) (Fig. 6c). This result is slightly different from the ranking of expression stability of all candidate reference genes in all samples by the other two software. BestKeeper analysis revealed SD values greater than 1 for *L25*, disqualifying them as RGs. The stability ranking results of BestKeeper for CRGs showed similarities that obtained from Normfinder and geNorm.

A comprehensive evaluation of the three methods was made by using refFinder to eliminate the deviation caused by a single method. According to the evaluation results, the expression of *GAPDH* and *NAPRT* was the most stable in Dp, while *GAPDH* and *EF1-alpha* are the most stable in Dt (Table 2). In all samples, *EF1-alpha* and *GAPDH* were also the most stable ones (Fig. 6d, Table 2). Moreover, *GAPDH* was the most stable according to the comprehensive ranking in all samples, while *L25* was the least.

### Minimum number of reference genes

**Figure 7:**
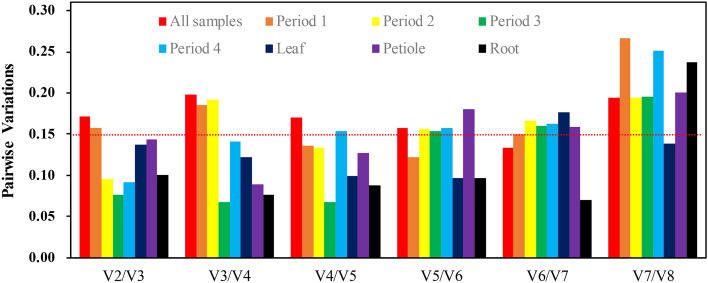
GeNorm calculated the minimum number of reference genes necessary for reliable and accurate normalization. Determination of the optimal number of reference genes for qRT-PCR normalization in all samples, different tissues and different periods.

To select the optimal minimum number of RGs for credible normalization, the paired variation value (V_N_/V_N +1_) was calculated by geNorm^27^. The best reference gene combinations in the gene expression studies of different tissues of *P. ternata* were *GAPDH* and *EF1*-alpha, and the best reference gene combinations in the gene expression studies of different periods of *P. ternata* were *GAPDH* and *NAPRT*. In the gene expression studies covering Dp and Dt of *P. ternata* (Fig. 7), V_6_/V_7_ was 0.134, less than 0.15, but V_2_/V_3_ (0.172) was close to 0.15. Considering the cost of the experiment, we believed that two RGs (*GAPDH* and *EF1-alpha*) were also competent for the calibration of these samples. Except for the first period, the values of V_2_/V_3_ in other Dp and Dt samples were all lower than 0.15 (Fig. 7), which suggested that the best reference gene combinations in Dp and Dt were *GAPDH*, *NAPRT* and *GAPDH*, *EF1-alpha*, respectively (Table 2).

### Validation of the selected reference gene expression

**Figure 8:**
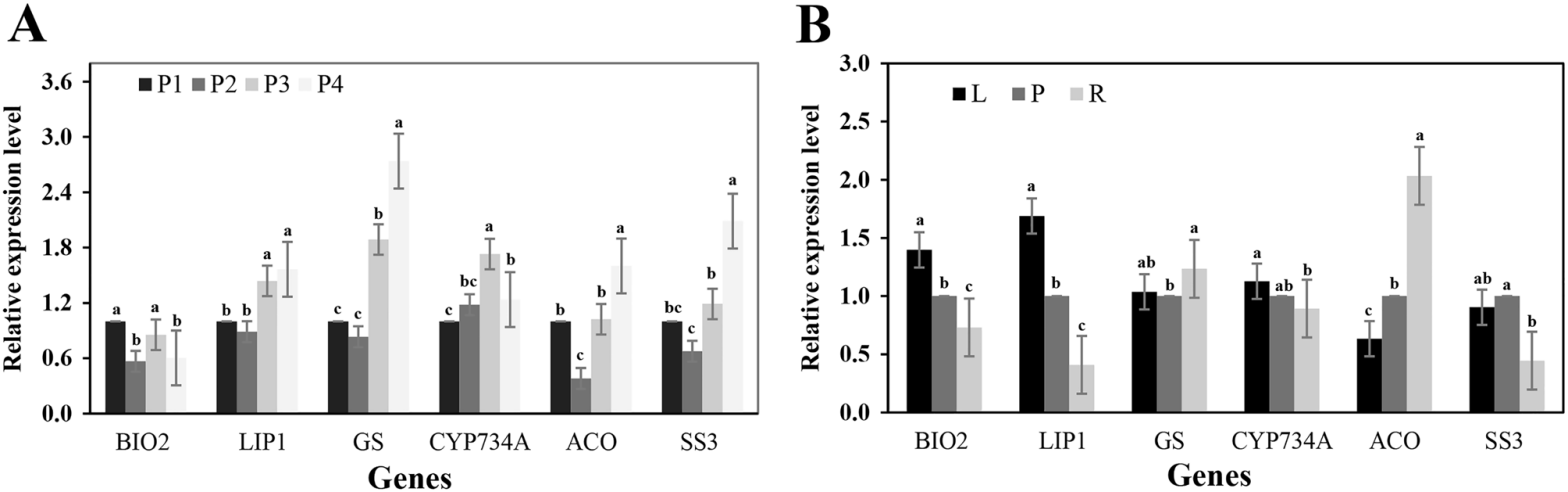
Relative expression levels of selected genes in different tissues and stages of bulblet development of *P. ternata*. The results were normalized using the selected stable reference gene in sample sets. The bars indicate the standard error (± SE) evaluated from three biological replicates. (**A**) Different periods. P1 = Period 1; P2 = Period 2; P3 = Period 3; P4 = Period 4. (**B**) Different tissues. L = Leaf; P = Petiole; R = Root. a, b, c, differences in expression levels of the same gene in different tissues and at different periods, according to the least significant difference (LSD) test *p* ≤ 0.01.

To evaluate the reliability of the most stable gene *GAPDH*, expression of potential bulbil development related genes in energy metabolism pathways and hormone response pathways were normalized. These genes have been obtained from transcriptome sequencing data (Table 3). In different periods (Dp), only *BIO2* showed a decreasing trend with the growth of *P. ternata* (Fig. 8A). However, with the growth of *P. ternata*, *LIP1*, *GS*, *ACO* and *SS3* showed a trend of decreasing firstly and then increasing trend in later periods (Fig. 8A). Only the *CYP734A* gene showed a trend of increase and then decreasing tendency with the growth of *P. ternata* (Fig. 8A).

**Table 3:** Delineations of six differentially expressed genes in bulbil formation of *P. ternata*.

Gene symbol	Homolog locus	Size(bp)	Annotation	Primer sequences (forward/reverse, 5′–3′)	Product size(bp)
*BIO2*	XM_015792856	1155	Biotin synthetase	TAGAAAAGCAACAAGCGGCG TGCCTCTCCAAGCCCAATTA	194
*LIP1*	XM_015777878	1176	Lipoic acid synthetase	TGTTGGGTTGTGGTGAGACT GAGGCCACATATCGGAAGCC	199
*GS*	XM_015770616	981	Glutamine synthetase	CGACACCACGGAGAAGATCA AGATGGCCTGGGGGTATAGG	194
*CYP734A*	XM_015764261	1581	Cytochrome P450 734A	GGATGTGCTTCCTGGGGTTT CAGTCGTGCCCTTTGACCTT	196
*ACO*	XR_003238359	912	1-Aminocyclopropane-1-carboxylateoxidase	CTGGTCGTCAACATCGGAGAT GACTGGTGGGTTGTCACTGG	192
*SS3*	XM_015780729	927	Starch synthase	TCTGACCAGCCGATTGTAGG GTCGAGCACGGTCACTATGG	199

In different tissues (Dt), P (petiole) was used as the control group and L and R as the experimental group to calculate the relative expression levels of the six genes. Among them, the expression abundance of *BIO2*, *LIP1* and *CYP734A* genes was the lowest in R and the highest in L, while the expression abundance of *ACO* was the lowest in L and the highest in R (Fig. 8B). The expression level of *SS3* in P was higher than that of L and R, while the expression level of *GS* was the lowest in P (Fig. 8B). The different and specific expression pattern conformed with the previous reports or the digital gene expression profile from our transcriptomic data^28^.

## Discussion

Although a great deal of research work has been done on tissue culture, identification and separation of active components and medicinal mechanism of *P. ternata*, there are still some problems in the production of *P. ternata*, such as low yield, continuous cropping obstacle and so on. It has a great influence on the medicinal resources of *P. ternata*^3^. To solve these problems, it is necessary to analyze the expression patterns of genes related to the development of *P. ternata*, then further explore molecular mechanism involved in it.

In the post-genomic era, qRT-PCR has become one of the essential technologies in gene transcription and functional genomics study, because of its high specificity and sensitivity. The study of gene expression profile can help us to understand the role of genes involved in a particular biological process^29^. qRT-PCR is often used to detect the expression level of low abundance mRNA and to verify the effectiveness of RNA interference in gene functional study^30^, and help us to further study the gene expression in plant growth and developmental processes, stress response, and other biological processes. However, it is essential to normalize the expression data of target genes with the help of RGs for a particular tissue type, independent of the developmental stage or experimental treatment conditions. The data obtained by using unverified RGs cannot be trusted^31^.The screening of various plant RGs has been widely carried out, but so far, it has not been found that a reference gene has a stable expression profile under all conditions of experimental^32^. Therefore, screening and evaluating the most stable RGs in specific research models were great significance to improve the accuracy of qRT-PCR experiments and clarify the scientific problems represented by qRT-PCR results in these models^33^.

Although the best RGs of many plants have been successfully screened under different experimental conditions, the RGs that can be stably expressed in different tissues and different developmental stages of *P. ternata* have not been reported. Eight CRGs were screened in present study. Among the candidate genes, *GAPDH*, *TUB*, *EF1-alpha* and *EF1-beta* are commonly used RGs in plants^16,34,35^, However, using *NAPRT*, *L25*, *PEPCK* and *ARL8B* as RGs in plants has not been well studied. In this study, the results indicated that common RGs (such as *GAPDH, EF1-alpha* and *TUB*) showed more stabilities than newly explored CRGs.

Discrepancy was observed in gene stability ranking and validation generated by the different algorithms (geNorm, NormFinder and BestKeeper). Thus, the refFinder algorithm was used to aggregate the evaluation results of the three algorithms to minimize the bias. The result of refFinder demonstrated that *GAPDH* was the most reliable reference gene in different periods and different tissues of *P. ternata* (Table 2). *GAPDH* was commonly used RGs in many species and tissues^36–38^.

The process of bulbil formation involved complex molecular mechanism, and plant hormones play a key role in the process, such as IAA, GA, ABA and ZR^39–41^. During the development of the bulbil, various nutrients continue to accumulate, providing nutrition supply for the mature bulbil to develop into a new individual. To verify the reliability of the best reference gene *GAPDH* in data normalization, and to explore the molecular mechanism of the occurrence and development of *P. ternata* bulbil, we screened six significant differential genes with potential role in the development of *P. ternata* from the early transcriptome data of *P. ternata*, and evaluated the relative expression levels of six genes in different tissues and different developmental stages of *P. ternata*. The purpose of this study was to lay a foundation for the follow-up study on the molecular mechanism of bulbil formation in *P. ternata*. *BIO2*(Biotin synthase), which catalyzes the conversion of desulfurized biotin to biotin in the process of biotin synthesis. Biotin is an essential micronutrient and a cofactor of several carboxylase involved in glucose, fatty acid and amino acid metabolism^42,43^. In different tissues of *P. ternata*, the expression abundance of *BIO2* in the aboveground part was higher than that in the underground part, showing a decreasing trend (Fig. 8B), which was consistent with the phenomenon that biotins were synthesized in the aboveground parts of *Arabidopsis thaliana* and then transported to roots by transporters to promote root growth^44^. *SS3* can encode a starch synthase, which not only participates in starch biosynthesis, but also negatively regulates instantaneous starch biosynthesis^45^. With the growth of *P. ternata*, the expression level of *SS3* gene increased as a whole, and the expression abundance of *SS3* gene was the highest in the fourth stage (Fig. 8A). In Dt, the expression abundance of *SS3* in petiole (P) was close to that in leaf, but higher than that in root (Fig. 8B). It was suggested that there was an increase trend of the synthesis of starch nutrients in petiole during development process, which accumulates nutrients for bulbil maturation.

*GS*, a glutamine synthetase, plays a critical role in the complex substrate of plant nitrogen utilization, and can protect leaves from excessive ammonia toxicity^46^. The expression level of *GS* increased with the development of *P. ternata*, and the expression level of *GS* in the fourth stage was much higher than that in the first stage (Fig. 8A), indicating that a large number of nitrogen-containing components such as amino acids, proteins and nucleic acids were needed in the process of *P. ternata* growth and bulbil development.

*CYP734A* was a member of the cytochrome P450 family^47^. In our previous transcriptome data, *CYP734A* was annotated into the zeatin biosynthesis pathway (ko00908), and the gene expression level was the highest in the third stage. Similar results were observed in present study (Fig. 8A). The ACC synthesized in the underground part of the plant, and then transported to the aboveground part, where it was converted to ethylene by ACC oxidase (*ACO*)^48^. In plant roots, exogenous ethylene can promote the initiation and elongation of root hairs^49^. Some studies have also found that endogenous ethylene can interact with other plant hormones to regulate the growth of plant taproots and further affect the absorption of nutrients^50^. In this study, the gene expression level of *ACO* in the underground part of *P. ternata* was much higher than that in the aboveground part (Fig. 8B), which needs further experimental study on the growth and development of bulbils in *P. ternata*.

## Conclusion

Based on the high-throughput transcriptome sequencing data and the statistics of commonly used RGs in plants, eight CRGs were selected and validated for qRT-PCR normalization in different tissues and four developmental stages in *P. ternata*. *GAPDH* was observed as the most stable reference gene in all samples. *GAPDH* and *EF-alpha* was the best combination for different tissues and developmental stage of *P. ternata.* The relative expression analysis of target genes that related to bulbil development, which selected from the transcriptome data of *P. ternata*, confirmed *GAPDH* was the best reference gene in developmental periods and different tissues of *P. ternata*. All the results of this study laid a foundation for the further study of the mechanism of bulbil formation of *P. ternata*.

## Materials and methods

### Plant materials

Seeds (bulbil) of *P. ternata* were collected from Qingshui County, Gansu Province (CHN, latitude 34.56 N, longitude 105.45 E) under the permission of the competent authority. All experimental procedures, including investigation and collection, were conducted under relevant institutional, national, and international guidelines and regulations. Professor Tao Xu (College of Life Sciences and Medicine, Zhejiang Sci-Tech University, China) identified the seeds of *P. ternata*. Seeds of *P. ternata* were stored in the medicinal plant germplasm resource bank of Zhejiang Sci-Tech University (CHN, latitude: 30.32 N, longitude: 120.35 E) with dry sand at low temperatures. *P. ternata* cultivar ‘0012’ was used as plant materials, and its seedlings were used to study the gene expression of *P. ternata* in different bulbil stages. Seeds were grown in a greenhouse of Zhejiang Sci-Tech University with a temperature range of 25 ± 1 °C and 12 h photoperiod. After germination, the seedlings of *P. ternata* were irrigated with water every three days and 1/5 MS solution once a week to maintain adequate moisture and nutrition. Referring to the research results of bulbil development by Luo et al.^51^, and according to our experimental settings, the leaves, petioles and roots of the four early stages of bulbil development were selected as samples (Fig. 1). All the harvested samples of *P. ternata* were immediately immersed in liquid nitrogen for freezing and stored at -80℃.

### RNA extraction and cDNA synthesis

Total RNA was extracted from 200 mg samples by using RNA prep Pure Plant Plus Kit (TIANGEN, China) refer to the manufacturer’s instructions. The purity and concentration of each RNA sample were examined using NanoDrop 2000 spectrophotometer (Thermo Scientific, China). The reverse transcription reaction was processed using *Evo M-MLV* reverse transcription kit II (ACCURATE BIOLOGY, China) in a 20 µL volume system containing 500 ng RNA followed the kit instructions.

### Candidate reference genes selection and primer design

According to the related research literature on the plant RGs^52–55^, *GAPDH*, *EF1-alpha*, *EF1-beta* and *TUB* were selected as a reference gene. Besides, *PEPCK*, *L25*, *ARL8B*, *NAPRT* were selected from transcripts data of *P. ternata* (Unpublished) as new CRGs (Table 1). Moreover, we identified the homologous nucleotide sequences of CRGs in *P. ternata* by BLAST. The primer of CRGs was designed using NCBI/Primer-BLAST (https://www.ncbi.nlm.nih.gov/tools/primer-blast/) based the following parameters: amplicon length of 180-200 bp; 18–22 bp of primer length; melting temperature (Tm) of 59–62 °C; GC content of 45–55%. All primer pairs of CRGs were synthesized by Youkang Biotechnology Company (Zhejiang, Hangzhou). Taq PCR Mix (2 ×) kits (Sangon biotech, Hangzhou, China) was firstly used for semi-quantitative RT-PCR to detect the specificity and validity of all primers. The amplified products of PCR were identified by 1% agarose gel electrophoresis and observed in gel Image System 1600 (Tanon, China).

### qRT-PCR conditions and Primer amplification efficiency

The reaction of qRT-PCR was preformed using SYBR**®** Green Premix *Pro Taq* HS qPCR Kit (Accurate biology, China) and run on 96-wells plates with the Applied Biosystems 7500 Real-Time PCR System (Applied Biosystems, CA, USA) in comparative C_T_ (ΔΔC_T_) of experiment type. PCR amplification system were prepared in 20 µL volumes containing:10µL of 2 × SYBR**®** Green Pro Taq HS Premix, 2µL of cDNA, 0.4µL of each primer, 0.4 μ L ROX reference dye, and 6.8 μ L ddH_2_O. The reaction conditions of qRT-PCR consisted of initial step of 30 s at 95˚C for pre-denaturation, and followed by 40 cycles at 95˚C for 5 s, 60˚C for 30 s. Subsequently, the melting curve of product was analyzed according to the default parameters of the instrument. Negative controls with RNase-free water instead of cDNA were included.

Standard curves from a serial dilution of pooled cDNA (mixture of leave, petiole and root; 1,10,102,103,104 × dilutions) were constructed to calculate the amplification efficiency of each pair of primers and the correlation coefficient (R^2^) estimates of each pair of primers calculated in Microsoft Excel 2019 using the equation E (%) = [10^(−1/slope)^ − 1] × 100^56,57^. The average cycle threshold (Ct) of each gene was derived from the three biological repeats, and each biological repeat was composed of three technical repeats.

### Analysis of gene expression stability

The stability of the expression of each candidate gene was analyzed using geNorm^23^, NormFinder^24^, BestKeeper^25^, and refFinder^26^. The analysis results were comprehensively evaluated using the comprehensive ranking platform refFinder (https://www.heartcure.com.au/reffinder/) combined with the geometric mean of the ranking to avoid the one-sidedness of a single analysis method.

### Validation of reference genes

Six genes related to the regulation of *P. ternata* bulbil growth were used to validate the selected *GAPDH*, the most stable gene in total samples of *P. ternata,* that determined by the refFinder algorithm. The reaction conditions and primer design parameters of qRT-PCR were consistent with those mentioned before. Information of six genes selected from the transcriptome of *P. ternata* was listed in Table 3 and Table S5. Formula 2^−ΔΔCT^ was used to calculate the relative expression levels of target genes^58^.

## Supplementary Information

Supplementary Information 1.

Supplementary Information 2.

## Data Availability

We confirm that all the data associated with this manuscript are freely available and are presented either within the main manuscript file or in the Supplementary Materials section.
