# Supplementary figures and images for: Selection of suitable candidate genes for mRNA expression normalization in bulbil development of Pinellia ternata
Source: Sci Rep. 2022 May 25;12:8849. doi: 10.1038/s41598-022-12782-5 (PMC9133075; doi:10.1038/s41598-022-12782-5)

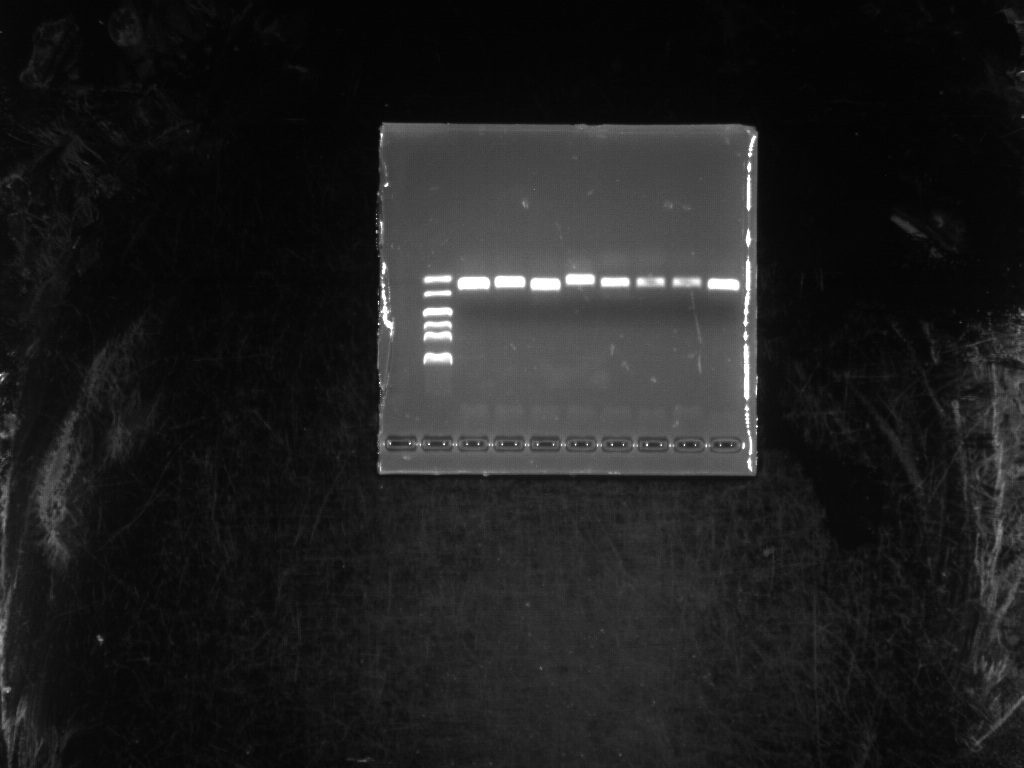

Supplement: Supplementary file 1 — Supplementary Information 1. [file 41598_2022_12782_MOESM1_ESM.tiff]
