# Supplementary material for: Selection of suitable candidate genes for mRNA expression normalization in bulbil development of Pinellia ternata
Source: Sci Rep. 2022 May 25;12:8849. doi: 10.1038/s41598-022-12782-5 (PMC9133075; doi:10.1038/s41598-022-12782-5)
Supplement: Supplementary file 2 — Supplementary Information 2. [file 41598_2022_12782_MOESM2_ESM.docx]

**Selection of suitable candidate genes for mRNA expression normalization in** ***Pinellia ternata***

Haoyu Fan^1^, Qiuling He^1,^ *, Yiheng Dong, Wenxin Xu, Yanlin Lou, Xuejun Hua, Tao Xu*

College of Life Sciences and Medicine, Zhejiang Sci-Tech University, Hangzhou, China

^1^These authors contributed equally to this work.

*Corresponding author

Email：xutao@zstu.edu.cn; qlhe@zstu.edu.cn

**S1 Table.** The raw Ct value of eight candidate reference genes in Leaf(L), petiole(P), and root(R) at different developmental periods (Dp) of *P. ternata*.

| **Samples**  **Gene** | **1-L** | **1-P** | **1-R** | **2-L** | **2-P** | **2-R** | **3-L** | **3-P** | **3-R** | **4-L** | **4-P** | **4-R** |
| --- | --- | --- | --- | --- | --- | --- | --- | --- | --- | --- | --- | --- |
| *EF1-beta* | 17.4 | 18.9 | 17.6 | 18.8 | 19.2 | 16.9 | 18.1 | 19.4 | 18.6 | 19.0 | 17.8 | 17.4 |
| *NAPRT* | 23.7 | 23.6 | 23.7 | 24.5 | 24.4 | 23.0 | 22.2 | 24.1 | 23.5 | 22.4 | 22.7 | 22.1 |
| *L25* | 26.4 | 30.4 | 25.8 | 27.7 | 29.1 | 27.7 | 27.6 | 28.3 | 30.7 | 27.8 | 31.3 | 28.2 |
| *PEPCK* | 20.4 | 20.1 | 19.1 | 21.2 | 20.7 | 18.6 | 20.2 | 21.8 | 19.6 | 20.4 | 19.8 | 17.6 |
| *GAPDH* | 17.2 | 18.6 | 17.5 | 17.3 | 18.3 | 16.8 | 17.1 | 18.5 | 17.9 | 17.8 | 17.6 | 17.2 |
| *TUB* | 17.3 | 18.4 | 19.0 | 18.7 | 17.9 | 18.5 | 18.7 | 18.7 | 20.1 | 18.5 | 19.3 | 18.9 |
| *ARL8B* | 17.6 | 18.4 | 18.8 | 20.5 | 17.8 | 17.8 | 17.5 | 18.6 | 18.5 | 18.0 | 19.7 | 17.7 |
| *EF1-alpha* | 16.8 | 18.8 | 18.0 | 17.3 | 19.0 | 17.2 | 17.1 | 18.5 | 18.1 | 18.2 | 18.0 | 17.3 |

**S2 Table.** Expression stability of eight candidate reference genes as calculated by geNorm.

| Rank |  | Dp | | | |  | Dt | | |  | All samples |
| --- | --- | --- | --- | --- | --- | --- | --- | --- | --- | --- | --- |
|  |  | P1 | P2 | P3 | P4 |  | L | P | R |  |  |
| 1 |  | *GAPDH*  0.82 | *NAPRT*  0.81 | *EF1-alpha* 0.62 | *NAPRT*  0.79 |  | *EF1-beta*  0.71 | *EF1-alpha*  0.6 | *GAPDH*  0.81 |  | *GAPDH*  0.84 |
| 2 |  | *EF1-beta*  0.85 | *GAPDH*  0.84 | *GAPDH*  0.63 | *EF1-alpha* 0.79 |  | *L25*  0.72 | *GAPDH*  0.61 | *EF1-alpha* 0.84 |  | *EF1-alpha*  0.9 |
| 3 |  | *EF1-alpha* 0.95 | *EF1-beta*  0.92 | *ARL8B*  0.65 | *GAPDH*  0.81 |  | *PEPCK*  0.72 | *EF1-beta*  0.62 | *EF1-beta*  0.89 |  | *EF1-beta*  0.92 |
| 4 |  | *ARL8B*  0.99 | *L25*  0.97 | *EF1-beta*  0.69 | *TUB*  0.98 |  | *GAPDH*  0.76 | *TUB*  0.64 | *NAPRT*  0.92 |  | *NAPRT*  1.07 |
| 5 |  | *NAPRT*  1.07 | *EF1-alpha* 1.03 | *NAPRT*  0.75 | *ARL8B*  1.04 |  | *TUB*  0.78 | *ARL8B*  0.7 | *TUB*  1 |  | *ARL8B*  1.13 |
| 6 |  | *TUB*  1.12 | *PEPCK*  1.07 | *TUB*  1.1 | *EF1-beta*  1.12 |  | *EF1-alpha* 0.79 | *PEPCK*  0.77 | *PEPCK*  1.06 |  | *TUB*  1.14 |
| 7 |  | *PEPCK*  1.28 | *TUB*  1.27 | *PEPCK*  1.28 | *PEPCK*  1.35 |  | *NAPRT*  1.21 | *NAPRT*  0.8 | *ARL8B*  1.12 |  | *PEPCK*  1.2 |
| 8 |  | *L25*  2.18 | *ARL8B*  1.64 | *L25*  1.59 | *L25*  1.77 |  | *ARL8B*  1.22 | *L25*  1.92 | *L25*  1.67 |  | *L25*  1.66 |

P1= Period 1; P2= Period 2; P3= Period 3; P4= Period 4; L=Leaf; P= Petiole; R=Root; All samples are a combination of different periods (Dp) and different tissues (Dt).

**S3 Table.** Expression stability of eight candidate reference genes as calculated by NormFinder.

| Rank |  | Dp | | | |  | Dt | | |  | All samples |
| --- | --- | --- | --- | --- | --- | --- | --- | --- | --- | --- | --- |
|  |  | 1 | P2 | P3 | P4 |  | L | P | R |  |  |
| 1 |  | *EF1-beta*  0.04 | *NAPRT*  0.172 | *GAPDH*  0.047 | *NAPRT*  0.08 |  | *PEPCK*  0.084 | *GAPDH*  0.196 | *EF1-beta*  0.016 |  | *GAPDH*  0.16 |
| 2 |  | *GAPDH*  0.04 | *GAPDH*  0.302 | EF1-alpha  0.047 | *EF1-alpha*  0.161 |  | *EF1-beta*  0.259 | *EF1-alpha*  0.225 | *TUB*  0.016 |  | *EF1-alpha*  0.28 |
| 3 |  | *EF1*-*alpha*  0.239 | *EF1-beta*  0.351 | *ARL8B*  0.064 | *GAPDH*  0.243 |  | *GAPDH*  0.323 | *TUB*  0.425 | *GAPDH*  0.109 |  | *EF1-beta*  0.31 |
| 4 |  | *ARL8B*  0.485 | *PEPCK*  0.517 | *EF1-beta*  0.217 | *ARL8B*  0.404 |  | *L25*  0.33 | *EF1-beta*  0.472 | *EF1-alpha* 0.161 |  | *NAPRT*  0.52 |
| 5 |  | *NAPRT*  0.52 | *L25*  0.532 | *NAPRT*  0.241 | *TUB*  0.417 |  | *TUB*  0.378 | *NAPRT*  0.5 | *PEPCK*  0.325 |  | *ARL8B*  0.56 |
| 6 |  | *TUB*  0.63 | *EF1-alpha*  0.606 | *TUB*  0.6 | *EF1-beta*  0.644 |  | *EF1-alpha*  0.413 | *ARL8B*  0.595 | *ARL8B*  0.357 |  | *TUB*  0.56 |
| 7 |  | *PEPCK*  0.684 | *TUB*  0.689 | *PEPCK*  0.854 | *PEPCK*  0.797 |  | *ARL8B*  0.753 | *PEPCK*  0.601 | *NAPRT*  0.45 |  | *PEPCK*  0.67 |
| 8 |  | *L25*  1.472 | *ARL8B*  1.065 | *L25*  1.074 | L25  1.178 |  | *NAPRT*  0.756 | *L25*  1.102 | *L25*  1.317 |  | *L25*  1.06 |

P1= Period 1; P2= Period 2; P3= Period 3; P4= Period 4; L=Leaf; P= Petiole; R=Root; All samples are a combination of different periods (Dp) and different tissues (Dt).

**S4 Table.** Expression stability of eight candidate reference genes as calculated by BestKeeper.

| Rank |  | Dp | | | |  | Dt | | |  | All samples |
| --- | --- | --- | --- | --- | --- | --- | --- | --- | --- | --- | --- |
|  |  | P1 | P2 | P3 | P4 |  | L | P | R |  |  |
| 1 |  | *NAPRT*  0.03 | *TUB*  0.27 | *ARL8B*  0.45 | *NAPRT*  0.17 |  | *GAPDH*  0.22 | *GAPDH*  0.32 | *GAPDH*  0.38 |  | *TUB*  0.47 |
| 2 |  | *ARL8B*  0.44 | *NAPRT*  0.7 | *EF1-alpha*  0.6 | *GAPDH*  0.21 |  | *PEPCK*  0.34 | *EF1-alpha*  0.32 | *EF1-alpha*  0.4 |  | *GAPDH*  0.48 |
| 3 |  | *PEPCK*  0.58 | *L25*  0.74 | *EF1-beta*  0.6 | *TUB*  0.26 |  | EF1-alpha  0.55 | *TUB*  0.5 | *ARL8B*  0.47 |  | *EF1-alpha*  0.6 |
| 4 |  | *GAPDH*  0.69 | *GAPDH*  0.75 | *GAPDH*  0.62 | *EF1-alpha*  0.34 |  | *TUB*  0.57 | *EF1-beta*  0.67 | *EF1-beta*  0.66 |  | *ARL8B*  0.67 |
| 5 |  | *EF1-beta*  0.76 | *EF1-alpha*  1.08 | *TUB*  0.73 | *EF1-beta*  0.86 |  | *L25*  0.58 | *NAPRT*  0.74 | *NAPRT*  0.66 |  | *NAPRT*  0.7 |
| 6 |  | *TUB*  0.76 | *EF1-beta*  1.47 | *NAPRT*  0.99 | *ARL8B*  1.26 |  | *EF1-beta*  0.81 | *ARL8B*  0.81 | *TUB*  0.71 |  | *EF1-beta*  0.73 |
| 7 |  | *EF1-alpha*  1.05 | *PEPCK*  1.9 | *PEPCK*  1.49 | *PEPCK*  2.14 |  | *NAPRT*  1.43 | *PEPCK*  0.99 | *PEPCK*  0.94 |  | *PEPCK*  0.86 |
| 8 |  | *L25*  10 | *ARL8B*  2.54 | *L25*  3.36 | L25  4.39 |  | *ARL8B*  2.56 | *L25*  2.41 | *L25*  10.16 |  | *L25*  1.31 |

P1= Period 1; P2= Period 2; P3= Period 3; P4= Period 4; L=Leaf; P= Petiole; R=Root; All samples are a combination of different periods (Dp) and different tissues (Dt).

**Supplementary materials: Partial mRNA sequences of genes used in this study.**

>OM324012. PEPCK

ATGTCCGCCAACGGCGAGTTCAGCTTCACCAAGGATGACGAGGCGACGTCCCCTGTCGCCGGCTCCGGCGGCGCGGGAAGGCCCGCGGGCCGGCCAGCGCTGGGCGTGGTGGTGACGAAGAAGAGCCTGCCGAAGATCCAGACGCACGCGAAGAAGGAGGACGACGGGATCTGCCACGACGACAGCACCACGCCGGTGAAGGCGCAGACCATCGACGAGCTCCACTTGCTGCAGAAGAAGAAGTCGGCGCCCACCACCCCCATCAAGAGCGCCCAGTCGGGGCCTTTCGCGCCGCCCATCTCCGAGGAGGATCGCCACAAGCAGCAGCTCCAGTCCATCAGCGCGTCGCTTGCGTCGCTGACGCGGGAGACGGGGCCGAGACTGGTGAAGGGCGACCCCGATCGGAAGGCGGCGGAGACGCCGCGGGTGGCCTCCCACTCCCACGACTACTTCGTCCCCTCCATCAGCATCAGCGACAGCGCCCTCAAGTTCACCCACGTCCTCTACAACCTCTCCCCCGCCGAGCTGTACGAGCAGGCCATAAAGTATGAGCGTGGCTCCTTCATCACGTCCAGCGGTGCCCTGGCCACCCTCTCCGGTGCGAAGACCGGGCGCTCTCCCCGGGACAAGCGCGTCGTCAGGGATGAGACCACCGAGGACGACCTCTGGTGGGGCAAGGGCTCACCCAACATCGAGATGGACGAGCACACCTTCATGGTGAACAGGGAGAGGGCGGTCGACTACTTGAATTCTCTGGACAAGGTCTTTGTGAATGACCAATTCTTGAATTGGGATCCCGAACATCGGATCAAAGTCCGCATCGTCTCTGCAAGAGCCTACCATTCTTTGTTCATGCACAACATGTGTATCCGACCCACACCTGAAGAGCTGGAGGATTTTGGTACTCCAGACTTTACGATATATAACGCAGGCCAGTTCCCTTGTAATCGTTATACACATTATATGACATCATCGACTAGCATAGATCTTAACCTTGCTAGAAGAGAAATGGTCATCCTTGGCACACAGTATGCCGGGGAGATGAAGAAAGGCCTGTTCGGCGTAATGCACTATCTCATGCCCAAGAAAGGGATCCTGTCCCTGCACTCTGGATGCAACATGGGGAAAGATGGAGATGTTGCACTGTTCTTTGGACTGTCAGGTACTGGGAAGACAACTCTGTCTACAGATCATAATAGGTACCTCATTGGAGATGATGAGCACTGCTGGAGTGACAACGGTGTTTCTAACATCGAAGGAGGCTGTTATGCGAAGTGCATCGACCTCTCAAGGGAGAAGGAGCCAGATATTTGGAATGCAATAAGATTCGGAACTGTACTGGAGAATGTGGTCTTTGATGAACACACCCGAGAAGTGGAATACTCAGATAATTCTGTCACAGAGAACACCAGGGCTTCCTACCCGATCGATTACATCCCGAATGCCAAGATACCATGCGTTGGGCCACACCCAAAGAATGTCATCCTTCTGGCATGCGATGCATTTGGCGTTCTCCCTCCTGTCAGCAAGCTGAGCTTGCCACAAACCATGTACCACTTCATAAGTGGCTACACAGCACTGGTTGCTGGCACGGAGGACGGCATCAGGGAGCCGCAGGCGACCTTCTCGGCCTGCTTTGGGGCCGCTTTCATAATGCTGCACCCCACGAAGTACGCGGCAATGCTGGCTGAGAAGATGCAGAAGTATGGAGCCACAGGATGGCTCGTGAACACTGGCTGGTCTGGGGGAAGGTATGGGGTTGGCAACCGCATCAAGCTGGCTTACACTCGTAAGATCATCGACGCCATCCACTCAGGTGACCTTCTGAAGGCGAGTTACAAGAAGACGGAGGTGTTTGGGCTCGAGATACCTACGGAGATCGAAGGTGTGCCGGCCGAGATCCTGAACCCTGTCAACACCTGGGCGGACAAGGAGGCATACAACGAGACCGTGCTGAAGCTGGCTGGCCTCTTCAAGAGGAACTTCGAGGTGTTCGCCAACTACAAGATTGGCACGGACGGCCAGCTGACCCAGGAGATCCTGGAGGCCGGTCCCATTTTCTGA

>OM324013. GAPDH

ATGACCGGGCGCAGCCGCGGGGGCGCCGACCATGGCGGCGGGGAGGAGAGGGGCCTCTTCTGGAAGCTCCCCGTCGTGAGGTCCAGGGAGCTCGGCAAGCTTGGCCCCGGCATGGGGCTGGGCGCCGGCTGCGGCGTGGGCTTCGGCCTCGGCCTCTTTGGAGGTGGGGGACTCGGAGTGGGGTTTCCTGGGTTGCAGTTCGGGTTTGGCGTCGGAGCGGGGTGCGGGATCGGCCTGGGGTTCGGCTACGGCGTTGGTAGAGGGGTGGCGGTGGACGAGCACCGGAGGCACTCCAATGTTGGGCAACCCATGGGAGCTCTCCCTTCACATGAACAGCTAGTCTCCTTGATGGATGAACTCGTCGAGAATGCGAAGAAACTGGTTAGAACCACCTCGAAAACCATTGAAAAATGGAGGTGA

>OM324014. TUB

ATGAGGGAGATCCTGCACATCCAGGCGGGGCAGTGCGGCAACCAGATCGGCGGCAAGTTCTGGGAGGTGGTCTGCGACGAGCACGGGATCGACGCCAGGGGCAACTACACGGGCGACTCCCACCTGCAGCTGGAGAGGGTGAATGTCTACTACAACGAGGCCAGCGGGGGGCGGTACGTCCCCCGGGCGGTGCTGCTGGATCTGGAGCCCGGCACCATGGACGCCCTCCGCACCGGGCCCTACGGCCAGATCTTCCGCCCCGACAACTTCGTCTTCGGACAGTCTGGTGCGGGAAACAACTGGGCAAAGGGCCACTACACTGAGGGAGCAGAGTTGATCGACTCTGTTCTTGATGTCGTGAGGAAGGAGGCTGAAAACTGTGATTGCCTTCAAGGTTTCCAAGTCTGCCACTCTCTTGGTGGGGGAACTGGTTCTGGGATGGGCACACTGCTGATCTCAAAGATTAGAGAGGAATATCCTGACAGGATGCTGCTCACATTCTCTGTTTTCCCCTCACCAAAAGTATCCGACACTGTGGTGGAGCCGTATAATGCAACTCTGTCCGTCCATCAACTTGTTGAAAACGCAGATGAGTGCATGGTCTTGGACAATGAGGCTCTTTATGACATCTGTTTCCGGACACTTAAGCTTACCACACCAAGCTTTGGAGACCTGAACCACCTGATCTCGGCGACCATGAGTGGCGTCACTTGCTGCCTCCGGTTCCCCGGCCAGCTGAACTCCGACCTCCGGAAGCTCGCCGTCAACCTGATCCCCTTCCCGCGCCTCCACTTCTTCATGGTGGGCTTCGCCCCGCTGACCTCCCGCGGCTCCCAGCAGTACCGGGCGCTGACCGTGCCGGAGCTCACCCAGCAGATGTGGGACGCCAAGAACATGATGTGCGCCGCCGACCCCCGCCACGGCCGCTACCTCACCGCCTCCGCCATGTTCCGGGGGAAGATGAGCACCAAGGAGGTGGACGAGCAGATGATCAACGTGCAGAACAAGAACTCGTCCTACTTCGTGGAGTGGATCCCCAACAACGTCAAGTCCAGCGTGTGCGACATCCCGCCGCGGGGCCTCTCCATGGCCTCCACCTTCATCGGGAACTCGACCTCCATCCAGGAGATGTTCCGGAGGGTGAGCGAGCAGTTCACGGCCATGTTCAGGAGGAAGGCCTTCTTGCACTGGTACACCGGGGAGGGCATGGACGAGATGGAGTTCACCGAGGCCGAGAGCAACATGAACGACCTGGTGTCGGAGTACCAGCAGTACCAGGACGCCACAGCCGACGAGGAAGGGGACTACGAGGAAGAGGAGCAGGAGGGCGCCGAGTACAACTAG

>OM324015. ARL8B

ATGGGGCTCTGGGAAGCCTTCCTCAACTGGCTGCGCAGCCTGTTTTTCAAGCAAGAAATGGAGCTTTCTTTAATAGGACTGCAGAATGCTGGAAAGACATCCCTTGTGAATGTTGTTGCGACTGGTGGCTACAGTGAAGATATGATTCCCACTGTAGGATTTAATATGAAGAAAGTCACTAAGGGTAATGTGACAATAAAGTTGTGGGACCTTGGAGGACAACCCAGGTTTCGCAGTATGTGGGAACGATATTGTCGTGCTGTCTCTGCTATTGTGTATGTTGTCGATGCAGCAGATCGTGATAACTTGCCCATCTCAAGAAGTGAACTACATGACCTGTTGANN

>OM324016. EF1-alpha

ATGAACAAGAGGTCATTCAAGTATGCTTGGGTGCTTGACAAGCTGAAGGCTGAGCGCGAGCGTGGTATCACCATCGATATTGCTCTCTGGAAATTTGAGACAACCAAGTACTACTGCACTGTTATTGATGCTCCTGGGCATCGTGACTTCATCAAGAATATGATCACTGGAACTTCTCAGGCAGATTGTGCTGTGCTAATTATCGATTCCACTACTGGTGGATTTGAAGCTGGAATTTCCAAGGATGGCCAGACTCGTGAACATGCCCTGCTTGCTTTCACTCTGGGAGTGAAGCAGATGATCTGCTGTTGCAACAAGATGGATGCCACAACCCCAAAGTATTCAAAGGCGAGATATGATGAAATTGTGAAAGAAGTGTCGTCCTACCTCAAGAAAGTGGGTTACAACCCGGACAAGATTGCATTTGTCCCCATATCTGGTTTTGAGGGCGACAACATGATCGAGAGGTCTACCAACCTTGACTGGTACAAAGGACCCACCCTGCTTGAAGCTCTGGACTTGATCCAGGAACCAAAGCGCCCCTCGGACAAGCCCCTTCGCCTTCCTCTGCAGGATGTTTATAAGATTGGAGGCATTGGCACAGTGCCCGTTGGTCGTGTTGAAACCGGAGTTCTCAAGCCTGGTATGGTTGTTACTTTTGGCCCCACTGGGCTCACCACTGAAGTTAAATCCGTGGAAATGCACCATGAATCTCTGCCAGAGGCCCTCCCTGGTGATAATGTAGGCTTCAATGTCAAGAATGTGGCAGTAAAAGATCTGAAGCGTGGTTTCGTTGCATCCAACTCCAAGGACGACCCAGCAAAGGAGGCTGCTAATTTCACCTCCCAGGTCATCATTATGAACCACCCGGGGCAGATTGGCAATGGTTATGCCCCAGTCCTTGATTGCCACACCTCCCATATCGCTGTGAAATTTGCTGAGCTGCTGACCAAGATTGACAGACGTTCTGGCAAGGAACTGGAGAAGGAGCCAAAGTTTTTGAAGAATGGCGATGCTGGGATGGTAAAAATGATTCCTAGTAAACCAATGGTGGTGGAAACATTCTCTGAGTACCCTCCGCTTGGTAGATTTGCCGTTAGGGACATGAGGCAGACGGTTGCTGTTGGTGTCATTAAGAGTGTGGAGAAGAAGGATCCATCTGGTGCCAAGGTGACCAAGTCTGCTGCAAAGAAGAAGTGA

>OM324017. EF1-beta

ATGGCTGCCACCTTCTCTGATCTGCACACCGACTCCGGCCTCAAGGCCCTCGATGAGTACCTTTCCGGCAGGACCTTCATCTCCGGAGATGAGATCAGCAGGGACGATGTGAAGGTCTACGCGGCCGTGCTGAAGAAGCCTGGTGACGGGTTCGCCAACGTGGGCCGCTGGTACGACAGCGTTGCTTCGGTGCTTGCTGCAAGGTTTCCAGGTGAGGCTGTTGGTGTCAGGGTTTCTGCAGGTGCAGCAGCTCCTGCACCTTCTGCAGAGGCAGTAAAGGATGCTCCCAATGAAGATGATGATGATTTGGACTTGTTTGGTGATGAGACTGAGGAGGAGAAGAAGGCTGCGGAGGAGAGGGAGAACTTTGCTCAACGCCTGTTTGTTTGCATGCCATCAGACTCAATACCAGGAGACAAGATTCATAGTGGCAAGTTTTCGGTTCTCATGGATGTGAAGCCTTGGGATGACGAGACGGACATGAAGAAATTGGAAGAGGCTGTTCGGAGCATTCAAATGCCCGGCCTTCTCTGGGGAGCATCCAAGCTCGTGCCCGTCGGCTACGGCATCAAGAAACTGCAGATCATGCTCACAATCGTGGATGACCTCGTGTCCGTCGACAGCCTTATCGAGGAACACCTGACAGTTGAGCCCTGCAACGAGCACATCCAGAGCTGCGACATCGTCGCCTTCAACAAGATCTGA

>OM324018. L25

ATGGCGTGGTGGGGCTGCGCCAGAGCCGGAGTGCGGCGGAGGTGGGAGGCCGCCGCCGCCGCCGCGTCCGCCCGGACATACTACACGATCCAGGCCGTTCCCAGGGAGTACACCGGCAGCAGGATAGCGGCCAAGGAGAGGGCCCTCGGCCGGATCCCGGCCGTGGTGATCGCGCAGGGCGGCGGGGACGGCCCCGAGGCCGCCAGGAAGCTGCTGGTCACCGCCGACAGCAAGCAGATCGTCACCCTGCTCAAGCGGGTGCCCTTCTTCTGCTCCACCACGTTCGTGCTCCAGCTCCGCGCCGGCGCCGGATCCTCCGCCGTCCTCCAATCTGGCACTGTTCTCCCCATAAAGGTTCACAAGGATGCCGAGACTGGGAATATCCTGAACTTGGTCCTTGCGTGGGCGGACAAGGGTACCGAGCTGAAGGTGGATGTGCCAGTCGTGTATAAAGGAGAGGACGTGTGCCCGGGTCTGCAAAAAGGAGGATATCTGCAGAAGATTAGGACCAGTGTAAAGTACCTGTGCCCAAGTGAGCATATTCCCCCAAAAGTTGAGGTAGACTTGGCAAAGCTGGACATTGGCGACAGGGTTCTTCTAACAGACATCGAAGTCGACCCATCACTGAAGCTCCTGAGTAAAAACGAAACCATGCCCATCTGCAAACTTGTGGCTACAAAGCCAGATGAAGCGAAACCGATCGTAGTTTCTGAACATACAGAAGAGGCTTCTGAACAACCCTGA

>OM324019. NAPRT

ATGGCTGCGGCGAACGGATCGATCCGGATGGATGGTGACGAGGACGGGGCGGCGGCGGCGCGCCACCGGATACCGGGGCCTACCAACCCCATGGTGACTCCGCTGCTCACTGACCTCTACCAGTTCACCATGGCCTATGCCTACTGGAAGGCCGGGAAGCACACCGAACGCGCCGTGTTCGATTTGTTCTTCCGGAAGAATCCGTTTGGTGGTGAATATACCATCTTTGGCGGTCTGGAAGAATGCATTCGGTTTATTGCCAATTTCAAATTTGTGGAGGAGGAGATCTCGTTTCTGCGCTCTGTGATGCCTTCGTGTGAGGATGCTTTCTTTGATTATCTTAAGTCTATTGACTGCACTGATGTTGAGGTTTATGCTATTCCTGAGGGTTCTGTTGTTTTCCCTAAGATCCCCTTGATGAGAGTAGAAGGGCCAGTTGCTTTGGTACAACTTCTGGAAACTCCATTTGTGAATCTTGTCAATTTTGCTTCACTGCTGACCACAAATGCTGCGAGACATAGATTTGTGGCTGGCAAGTCTAAACATTTACTTGAGTTTGGGCTTCGACGGGCACAGGGACCTGATGGTGGAATCAGTGCATCAAAATATAGCTACATGGGTGGATTTGATGCAACAAGTAATGTAGCAGCTGGAAGTCTATTTGGAATACCACTTCGTGGAACCCATTCTCATGCTTTTGTTAGCTCATTTCTGAGCCCAGATGAGATCATTGATAAGGCCCTCAAAAGTCATGATGGTTCACACATTTGTGAAGATTTTGTTAGTTCGGTACAGACATGGCTCAGCAAAATTCAATGGTCAGATTCATTCCGTGGCATTTTTGGAGAGACTAATCAAAGTGAGCTGGCAGCATTCACCTCATATGCATTAGCATTCCCAAATAACTTCCTAGCGCTTGTAGACACATACGATGTTATGAAGAGCGGGATTCCTAATTTCTGTGCAGTAGCATTAGCACTTAATGACTTGGGGTACAAAGCATCTGGAATCAGGTTGGACTCTGGTGATCTAGCATATCTCTCCATTGAGGCACGGAAGTTTTTCTGTGCTATAGAGAAGGAATTCGACATTCCTGATTTTGGAAAAATGAATGTTACTGCTAGCAATGATCTTAATGAGGAAACACTTGATGCATTGAACAAGCAGGGACATGAAGTGGATGCCTTTGGTATTGGCACCTATCTTGTTACATGCTATGCTCAGGCAGCGCTTGGTTGTGTTTTCAAGCTCGTGGAGATCAATAATCAACCCCGTATGAAACTTTCCGAGGATGTTACCAAGGTTTCCATACCATGTAAAAAACGAATTTACAGATTGTATGGAAGAGAAGGCTACCCGTTAGTTGACATTATGACTGGAGAAAATGAACCACCACCAAAGGTAGCAGAAAGAATTCTCTGTCGTCATCCATTCAATGAATCCAAGAGAGCTTATGTTGTGCCACAGCATGTTGAAGAGCTTTTGAAGTGTTATTGGCCAGGTGATTCATGTAAATCAAGAGAAGAACTACCTACTCTGCAGAAGATCAGAGATCGCTGTATTCAACAGCTGGACCGTATGCGCCGAGATCACATGAGAAGACTAAACCCTACGCCTTACAAGGTCAGTGTGAGCGCGAAGTTGTATGACTTCATTCATTTCCTGTGGCTTAATGAGGCGCCAGTGGNN

> OM324020. BIO2

ATGCTGCTGATCCGATCCCTGCGCTTGCGCCTCTACACCCCCGCCTCCAGCTCCGCCTTCTCCTCCTTGGCGGCGGCGGCGGCGGTGCCGGCCGCCAGCGCCGCGTCCGCCGCGGCCGTGCAGTCGGAGAGGACGATACGGGAGGGGCCGAGGAACGACTGGAGCAGGGAGGAGATCAAGGCGATCTACGACTCCCCCATCCTCGATCTCCTATTCCACGGCGCTCAAGTTCATAGGCATGCCCAGAAGTTCCGGGAAGTACAACAATGTACTCTGCTTTCTATTAAGACAGGTGGATGCAGTGAAGACTGCTCATATTGTCCACAATCCTCTCGGTATGATACAGGACTGAAAGCTCAGAGGCTGATGAACAAGGATGCTGTCCTGCAAGCAGCAAGAAAGGCCAAAGATGCTGGCAGTACACGTTTTTGCATGGGTGCTGCATGGAGAGATACAGTTGGCAGGAAGACAAACTTTAACCAGATTCTCGAATATGTCAAGGAAATAAGAGAGATGGGCATGGAGGTATGTTGCACTTTAGGCATGCTAGAAAAGCAACAAGCGGCGGAACTAAAGAAGGCAGGCCTGACAGCATATAATCACAATCTTGACACCTCGAGAGAGTATTATCCCAGCATTATAACCACAAGAAGCTACGATGAAAGGTTGGAAACTCTTCAGTTTGTTCGTGAAGCAGGCATAAGCGTTTGCTCAGGAGGAATAATTGGGCTTGGAGAGGCAGAAGAAGATCGTGTTGGACTGTTGCATACATTAGCCACTCTCCCTACACACCCAGAGAGTGTTCCCATCAATGCACTCGTACCAGTCAAGGGCACACCCCTTGAGGATCAGAAGCCAGTTGAGATATGGGAGATGATTCGGATGATAGCAACAGCACGCATTGTCATGCCAAAAGCAATGGTGAGGTTATCTGCAGGACGGGTACGGTTCTCCATGTCCGAGCAAGCATTGTGTTTCCTAGCTGGGGCAAATTCCATCTTCGCAGGGGAGAAGCTGTTGACAACTCGAAACAATGATTTTGATGCTGACCAATTGATGTTCAAGGTTCTAGGGCTGGTCCCAAAGGCCCCCAGTTTTTCAGATGAAGGGGCAGAATTGGAAAACGAGGCATGTGAAGCAGCAGCTTCCAGTTAA

>OM324021. LIP1

ATGCATCTTCGCCTCATCCCCCTCGCCAGGGCCCTCCGGCGGCCCTGCTCCTCCCCATCCCCTTGCAGAGGCCTTTCTTCCTCGCCGGTCCTTCGCCAGCCAGATGTGGCACCGGCCGCCGCACCTGCAGGGCAGCCTCCGGCGCGGACGCTCGCGGAGCTGCGGCGCCGCCTGGCGGAGGAGGTCCCGACGCTCTCGGACTTCGCCTACTCCGTCGAGGTGGGGACGAAGAAGAACCCCATCCCCAAGCCCCAGTGGATGAAGCTGACCTATCCCGGGGGATCCAAGTACACCGCCATCAAGGCGAAGCTGCGGCAGCTGAAACTGCACACGGTGTGCGAGGAGGCCAAGTGCCCCAACCTCGGGGAGTGCTGGTCCGGCGGGGAGACGGGCACCGCCACTGCCACCATCATGATCCTCGGGGACACCTGCACCCGCGGATGCAGGTTTTGTAATGTCAAGACGTCAAGGACGCCTCCTCCTCCTGATCCAGATGAACCATCTAATGTTGCAGAGGCCATTGCATCCTGGGGTTTGGACTATGTTGTAATTACTAGTGTTGACCGTGACGACTTGGCTGATCAAGGAAGTAACCATTTTACTGAAACAATACATAAGCTGAAGGCTTTGAAGCCAGATATACTAATAGAAGCACTTGTTCCTGATTTTCGAGGTGACTCTGAATGTGTGGAAAAAGTTGCAAAGTCTGGATTGGATGTTTTTGCTCACAACATTGAGACAGTGGAAGAGTTGCAGTCTTCAGTTCGAGATCACCGTGCTAACTTCAAACAGTCCATTGAAGTTCTTAAAACAGCGAAGGACTATGCACCTATGGGTACTCTAACAAAGACGTCGATAATGTTGGGTTGTGGTGAGACTCCTGATCAGGTACTAAGCACAATGGAGAAAGTAAGGGCTGCAGGGGTTGATGTGATGACTTTTGGACAGTATATGAGACCATCAAAGAGGCATATGCCCGTCTCAGAGTATATCACACCAGAAGCTTTTGAGAGATATCGTTCAATTGGCATGGATATGGGCTTCCGATATGTGGCCTCGGGCCCGATGGTCCGATCCTCCTACAAGGCGGGAGAGTTCTACATCAAGTCCATGATTGAAGCCGATCGGGCGGCGACGGCGGAGGCGCCGTCTCCGTCGGCATCACCGGCTCCCTGA

>OM324022. GS

ATGTCTCTGCTCAACGATCTCGTCAATCTCAACCTCACCGACACCACGGAGAAGATCATCGCAGAGTACATATGGATCGGTGGATCGGGCATGGACATGAGGAGCAAAGCCAGGACCCTCCCAGGGCCGGTGACTGACCCGAAGGAGCTGCCCAAGTGGAACTACGATGGATCGAGCACCGGGCAGGCCCCCGGCGAGGACAGCGAGGTCATCCTATACCCCCAGGCCATCTTCAGGGATCCTTTCAGGAGGGGGAACAACATCCTGGTCATCTGCGACGCATACACCCCAGCTGGTGAGCCGATCCCCACCAACAAGAGATTTGCGGCCGAGAAGATCTTTAGCCACCCAGATGTTGTCGCCGAGGAGCCATGGTACGGCATTGAGCAGGAGTACACTCTCCTGCAGAAGGATGTGCATTGGCCTCTTGGATGGCCTGTTGGCGGCTTCCCCGGTCCACAGGGCCCATACTACTGTGGGGCTGGAGCTGACAAGGCATTTGGAAGGGACATTGTGGACTCCCATTACAAGGCATGCCTTTACGCTGGAATTAACATCAGTGGAATCAATGGTGAAGTTATGCCTGGTCAGTGGGAATTTCAAGTAGGACCTGCAGTTGGAATTTCTGCTGGTGATGAATTGTGGGTAGCACGGTACATCCTGGAGAGGATAACTGAGATTGCAGGCGTTGTCCTTTCGTTTGACCCAAAACCAATCCAGGGTGATTGGAATGGTGCTGGTGCTCACACCAACTACAGCACAAAGTCTATGAGGAACGATGGCGGCTTTGAAGTGATCAAAAAGGCAATTGAAAAGCTGGGCCTGCGTCACAAGGAACACATTGCTGCATACGGAGAGGGGAACGAGAGAAGATTGACCGGTCGACATGAAACTGCAGATATCCATAAATTCTCATGGGGAGTTGCAAACCGTGGTGCTTCTGTCCGTGTTGGCCGCGACACTGAGAAGGCTGGAAAAGNN

>OM324023. CYP734A1

ATGAGTGCGGCGGAGGAGGTCGGTGTGGTGTGGCGCACGAGGTGGGCCGTCTTCGCCTGGATGTGCTTCCTGGGGTTTCTGGGCGCCGCCCTGTGCTTCGTCTACGATGTGTTCTGGGCGAGGCCGGAGAGGATGAGGGCGAAGCTGAGGCAGCAGGGCATAAGGGGCCCGCCCCGCACGCCGTTCGTCGGCAACACACTGGATATGAAGAGGATCCAGGAGGAAGAGAAGCTCAGCAAGGTCAAAGGGCACGACTGTGCGGCCATTGTTTTGCCCTTTTTGGATCGTTGGAGGAAGGAATACGGTTCCATCTTCGTGTGCTCCATGGGAAGCTTGGAGATACTGTATGTGTGTGATGTGGGCATGGTGAGGGAGATCAACCAGTGCAAGTCCCTGGAACTGGGGAGACCCACCTACTTGCAGGCACTGTTCAGGCCTCTCTTTGGGCGTGGTATTATTTCTTCCAATGGGTCCACATGGGCCCACCAGAGGAAGGTCATTGCTCGGGAATTCTTCATGGACAAGGTCAAGGGCATGGTGGAGCTTATGGTGGAGGCCACCAAGCCAGTGTTGGAGTCATGGGAAACCAGGGTTCAATGCGAGGGGAACACTGCAGAAATAACTGTTGATGAGGACTTGAAAAATTTGTCTGCAGATGTGATCTCCAGAGCTTGCTTTGGGAGCAGCTACTCTGAAGGAAAAGAGATCTTTGTAAAGTTGCAGGCACTTAAGGTAGCCTTATCAAAGAATATTGTATTCCTTGGCATCCCTGGCATGAGATACCTCCCCACAAGTAACAACAGAGAGATCTGGAGGTTGGAACGTGAGAGCCAGGCATCGATCCTGGAAATAGTGAAGAAACGTCAAGAGGAACCAGAATCTTCATCTGAGAACCTACTTCAGTCTCTCCTTGAAAGTAGTGGTGATAGTTGTATAGATCCAGACAGCAGGGAAGATTTTATTGTGGACAACTGCAAGAATATCTATTTTGCTGGCCATGACACAACTGCAACATCTGCTGCTTGGTGTTTGATGCTACTCGCTTCACATCCAGAGTGGCAAACTCGAGCTCGTGATGAGGTGGTGGAGATCTGTGGAGACCAGAGTCCAACTGCTGATACACTTAGCAAGATGAAAACATTGACAATGGTGATCCAAGAAACTTTACGACTTTACCCGCCGGCAATCTTTATATCAAGAGAGGCATCTAAAGATATAAAGTTGGGAGATATCCATATCCCCAGAGGCCTGGCCGTTCGAATCCCAACATCAGCACTGCAGAGTGACCCAGAAATTTGGGGACCAGATGCATATGAGTTCAATCCAGAGCGGTTTGCTCACGGTATATCTGGAGCTTGCAAAAACCCTCAGGCTTACATCCCTTTCGGCACTGGGATTCGGACATGCATTGGACAAAACTTTGCCATGGTGGAGTTGAAGGTTATCCTCTCCCATATCTTGTCCAAATTTTCATTCTCTCTGTCCCCAAACTACTGCCATTCTCCGGCATTTAGATTGACATTAGAACCAGAGTTTGGTGTGCCTCTCATATTGAAGAAGGCACAACCCAGGAATCTATAA

>OM324024. ACO

ATCCCCGTCGTCGACCTCGGCGGCGCCGACGACCCCGTCCGGCGCCGGCGGGTCGTGGAGGAGGTGAGGCGGGCCTCCGAGACGTGGGGCTTCTTCCAGGTGGTGAACCACGGCGTCCCCGAGGGCGTGCTGGAGGAAGCCCTGCGGCGGGGGAGGCAGTTCTTCGAGCAGGACAAGGAGGCCAAGGCCGGGTACTACATGCGGGAGCCCGGGAAGAAGGTGTCCTTCTACAGCAATTTCTTCCTGTACAGCCCCCATCCGGTCAACTGGAGGGACAGCCTCACCTTCACCATGGCCCCGGAGCCCGCCGCCGCCGAGGAGATGCCCCCTGTGTGCAGGGACGCCGTCGTGGAGTACTCGGGGTACATGGGGAAGCTGGGGAGCTTGGTGTTCGAGCTGCTGTCGGAGGCGCTGGGTCTGGAGCCCGGCCGCTTGGAGGACATAGGCTGCGCCGAGGGGCGCAGCGTCCTGATCCACTACTATCCGGCGTGCCCGCAGCCGGAGCTCACCATCGGAACCTGCAAGCACGCCGACCCCGACTTCATGACCATCCTCCTGCAAGACGACATCGGAGGTCTGCAGGTCCTGCACGAGGGTCGCTGGGTGGACGTCTCCCCGCTCCCCGGAGCTCTGGTCGTCAACATCGGAGATCTTCTGCAGCTAATATCCAATGACAAGTTCCAGAGTGTTGAGCACAGAGTGTTGGCGAACAGTGTGGGTCCACGGATGTCAGTGGCATGCTTCTTCGTGGCTCTTAATCCCCCGTCTACAAAGATCTACGGTCCCATCAAGGAGCTGCTCTCCAGTGACAACCCACCAGTCTATCGAGAGACTTCGGTTAAGGACTTCGCTGCTTACTACACTGTCAAGGGGCAAAATGGGCAGTCTGGTCTGCCTCATTTCAAGCTGTGA

>OM324025. SS3

NAACACTACAAACATTATGGACTGAGTGATGCTCGAGTTATTTTCACAATTCACAATCTTGAGTTTGGGGTGCACTTCATTGGAAAAGCAATGACACATGCTGACAAGGCTACAACTGTTTCTCAAACTTACTCGAGAGAGGTTTCTGGAAATCCAGCTATTGCTCCACATCTTCATAAGTTCCATGGCATTGTGAATGGGATAGATCCTGATATATGGGACCCATACAATGACAACTTTATTCCTGTACCATATACATCTGATAATGTTGTGGAAGGTAAAAAGGCTGCCAAGGAAGCCCTGCAGCAAAGACTTGGCCTTAAGAGATCTGACCAGCCGATTGTAGGCATCATTACTCGTTTAACAGTCCAGAAGGGAATTCACCTCATTAAGCATGCCATTTGGCAGACCCTTCAACGTAATGGACAGGTCGTATTGTTGGGTTCAGCACCAGATCCTAGAATACAGAATGACTTTACTAATTTGGCAAATCAATTGCACTCTTGCCATAGTGACCGTGCTCGACTTTGTCTAACCTATGATGAACCACTTTCTCATATGATATATGCTGGAGCGGACTTTATTCTTGTCCCGTCAATATTTGAGCCTTGTGGACTTACTCAACTTATAGCTATGCGATATGGCTCTATACCAGTCGTTCGGAGAACTGGAGGACTATATGACACGGTATTTGATGTTGACAATGACAAAGAAAGAGCTCAAGCACAAGGTCTAGAGCCAAATGGTTTTAGCTTTGATGGAGCTGATGCTGCAGGTGTAGACTATGCTCTTAACAGAGCACTCTCTGCCTGGTATGATGGTCGAGATTGGTTCAATTCATTGTGTCGGCGGGTGATGGAGCAAGACTGGTCGTGGAACCGCCCTGCCCTTGATTACATGGAGCTCTACTACTCCGCCCGCAAGTAA
